# Characterisation of weak magnetic field effects in an aqueous glutamic acid solution by nonlinear dielectric spectroscopy and voltammetry

**DOI:** 10.1186/1477-044X-2-8

**Published:** 2004-11-30

**Authors:** Alexander Pazur

**Affiliations:** 1 Department Biologie 1 Universität München-Bereich Botanik, Menzingerstr. 67, D-80638 München, Germany

## Abstract

**Background:**

Previous reports indicate altered metabolism and enzyme kinetics for various organisms, as well as changes of neuronal functions and behaviour of higher animals, when they were exposed to specific combinations of weak static and alternating low frequency electromagnetic fields. Field strengths and frequencies, as well as properties of involved ions were related by a linear equation, known as the formula of ion cyclotron resonance (ICR, abbreviation mentioned first by Liboff). Under certain conditions already a aqueous solution of the amino acid and neurotransmitter glutamate shows this effect.

**Methods:**

An aqueous solution of glutamate was exposed to a combination of a static magnetic field of 40 μT and a sinusoidal electromagnetic magnetic field (EMF) with variable frequency (2–7 Hz) and an amplitude of 50 nT. The electric conductivity and dielectric properties of the solution were investigated by voltammetric techniques in combination with non linear dielectric spectroscopy (NLDS), which allow the examination of the dielectric properties of macromolecules and molecular aggregates in water. The experiments target to elucidate the biological relevance of the observed EMF effect on molecular level.

**Results:**

An ion cyclotron resonance (ICR) effect of glutamate previously reported by the Fesenko laboratory 1998 could be confirmed. Frequency resolution of the sample currents was possible by NLDS techniques. The spectrum peaks when the conditions for ion cyclotron resonance (ICR) of glutamate are matched. Furthermore, the NLDS spectra are different under ICR- and non-ICR conditions: NLDS measurements with rising control voltages from 100–1100 mV show different courses of the intensities of the low order harmonics, which could possibly indicate "intensity windows". Furthermore, the observed magnetic field effects are pH dependent with a narrow optimum around pH 2.85.

**Conclusions:**

Data will be discussed in the context with recent published models for the interaction of weak EMF with biological matter including ICR. A medical and health relevant aspect of such sensitive effects might be given insofar, because electromagnetic conditions for it occur at many occasions in our electromagnetic all day environment, concerning ion involvement of different biochemical pathways.

## Background

Weak magnetic fields and extremely low frequency electromagnetic fields (EMF) are omnipresent in natural environmental and increasingly man-made factors. A possible influence on life processes was already mentioned in the late 19^th ^century [1]. It is now recognized, that many organisms are capable of perceiving such fields, while less is known on the elementary perception. Three types of mechanisms are considered therefore, the orientation of ferromagnetic particles in tissues [2], singlet-triplet mixing states of macromolecules building radical pairs [3], and the ICR, whose persistent investigation began with the works of Liboff [4].

Ferromagnetism has been implicated in animal navigation (e.g. compass mechanism of migratory birds [5], and the magnetotaxis of certain bacteria [6]. The radical pair mechanism is independent of ferromagnetism and has putatively a higher magnetic sensitivity. It has been primarily studied in photosynthetic reaction centers and the respiratory chain [7], where triplet yields are modulated by electromagnetic interaction with fields as low as about 50 μT [8]. Already two decades ago effects were described by Blackman *et al. *[9], and later by [10-12], which require a combination of static and alternating magnetic fields. It turned out, that the magnetic field strength **B **of the static component and the frequency *f *of the alternating EMF relate to the "ion cyclotron resonance (ICR) formula":



whereas *m *is the mass and *q *the charge of ions involved. The explanation of the mechanism of this effect in an aqueous, more or less viscous environment seems to be difficult, nevertheless there are some efforts. Liboff [13] suggested that magnetic fields can interact in a resonant manner with endogenous AC electric fields in biological systems, instead of a direct interaction with external AC magnetic fields. Binhi [14] reviewed the mechanisms of magnetobiological effects, and tried to estimate the sensitivities and involved molecular topologies. Adair [15] questioned a model involving altered transition rates of excited ions by weak EMF, while others [16] consider the ionic environment, eg. properties of the water, with Ca^2+ ^as the most investigated ion. An altered Ca^2+^-transport was found in human lymphocytes [4]. The motility of benthic diatoms is effected, if ICR conditions are matched for Ca^2+ ^and K^+ ^in the range of 8–64 Hz, and static field strengths comparable to geomagnetic fields [17]. The germination rate of *Raphanus sativus *was altered, when the ICR conditions for Ca^2+^, K^+ ^and Mg^2+ ^were applied to the seedlings [18]. ELF effects on macromolecules indicate an ICR effect possibly caused by additionally involved alternating electric fields [19]. It is noteworthy remarkable that ICR conditions can be matched by combinations of the local geomagnetic field and man-made electromagnetic fields, especially the frequency range of power lines (50 or 60 Hz). Liboff *et al. *[20] suggest to consider ICR effects for the evaluation of epidemiological childhood leukaemia studies. The assessment of elevated brain cancer risk has been evaluated by Aldrich *et al. *[21] on the assumption of interactions of the geomagnetic field and a 60 Hz field component from power lines.

NLDS was developed during the past decade in order to investigate dielectric properties of small particles in aqueous solutions, using relatively simple electrochemical equipment. In the simplest case, a sinusoidal alternating electric field is applied to the solution by 2 electrodes, using peak to peak voltages up to 1.5 V and frequencies of 1 to 1000 Hz. Particles with a dielectric constant different from that of their environment (generally water) distort the field. This induces alternating voltages over and currents through the solution, which are detected by 2 auxiliary electrodes in order to avoid polarisation effects. Phase shifts and distortions of the obtained signals, as compared to the input signal, contain information on damping and relaxation kinetics. Therefore, the signals are *Fourier*-transformed and evaluated as power spectra in the frequency domain [22-24]. Usually, the sample is compared to a reference, which lacks the solute, but otherwise is identical. Sample and reference can either be measured one by one in a single chamber device, or simultaneous with a "dual-chamber" setup, which also needs a two channel data acquisition, and allows a real-time differential-NLDS (DNLDS). The data are usually calculated using the decibel (dB) scale for the intensity (power) *P_n_*:



Where U(n)_sample _is the signal output intensity of the n^th ^harmonic from the sample measuring channel, and U(n)_ref _the corresponding value from the reference channel.

Zhadin *et al. *[25] reported the alteration of electric properties of an electrolyte under ICR conditions. They found an increasing ion current through an aqueous glutamic acid (Glu) solution in narrow frequency bands (resonance), which could be described by equation (1). These results are the starting point for the present work, which is aimed to further elucidate this conduction mechanism. The influence of the concentration of Glu has been investigated, and the time resolved electric current through the solution is analyzed using "non linear dielectric spectroscopy" (NLDS), which indicate microcolloidial properties of the solvent-solute system. The NLDS was amplified by two features: The option of simultaneous data acquisition in two cuvettes (DNLDS), and the frequency resolved voltammetry (FRV), whereby simultaneous a AC voltammetry is performed [26]. By recording NLDS spectra at varying electrode voltages from e.g. 100–1100 mV, additional information was obtained on redox potentials. The electrode current never increases proportionally with the applied voltage but remains constant in the range of the counter voltage to an existing redox potential given by the investigated electrode-electrolyte system. This was used to improve the method by recording differential spectra (DNLDS). The integral over the spectrum represents one data point of a simple (not frequency resolved) AC voltammetry, while the intensity course of corresponding spectral data points provide information about the dielectric state of the redox reaction, e.g. its capacitive, time-dependent properties.

## Methods

### Preparations

All preparations were performed with doubly de-ionized water. The solutions were degassed and stored under Argon, in order to avoid oxidation of the solute and increased electrode fouling during the subsequent measurements. An acidic solution of 2.24 mM Glu was adjusted to pH = 2.85 ± 0.03 with a stock solution of 5 mM HCl. Equilibration was assumed, when the pH varied less than ± 0.03 for at least one minute. All procedures were performed at 20°C. For yielding a reference signal, an aqueous solution of HCl was provided by diluting the HCl stock solution with water to pH = 2.85. All solutions were stored at 4°C under Argon.

### Apparatus

**Figure 1:**
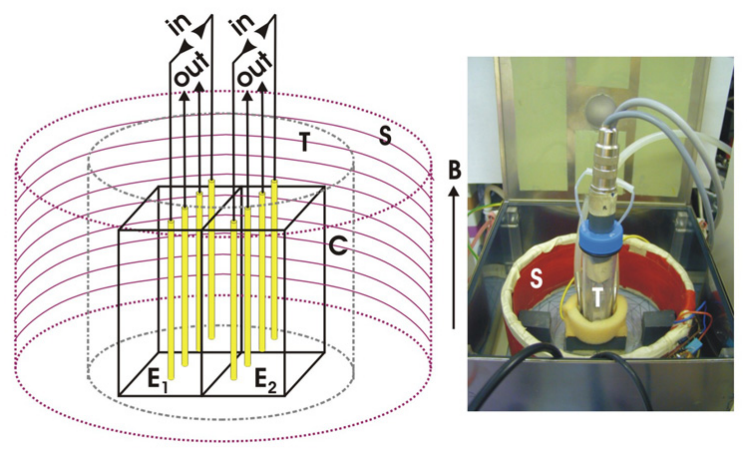
**Experimental facility. **Schematic sketch of the arrangement for the differential NLDS (DNLDS) experiments (left, components not drawn to scale) and photograph of the opened permalloy shielding box with the assembled sample carrier (right): Two arrays of 4 gold electrodes (**E_1_**, **E_2_**, length 10 mm, distance 2 mm) each are located in two adjacent perspex cuvettes (**C**) of 1 × 1 × 4.3 cm, enabling simultaneous acquisition of two liquid samples (used volume 1 ml each) under the same environmental conditions. The cuvettes are enclosed by a tank (**T**) for providing an Argon protection gas atmosphere. This all is mounted on a socket housing water temperature control and magnetic field monitoring, and is centered inside a cylindrical solenoid (**S**) consisting of 2 coils with a inner diameter of 16 cm and a height of 7 cm for independent generating the static and the alternating magnetic fields of vertical direction (**B**). The input signal to the sample is applied by the electrodes labeled "in", the probe signals are taken by the electrodes labeled "out" and connected to preamplifiers with symmetric inputs. The complete arrangement is enclosed by a shielding box of 1 mm Permalloy, which is bonded inside with perspex.

The experimental arrangement for differential non-linear dielectric spectroscopy (DNLDS) is shown in Figure 1. It allows the simultaneous evaluation of a sample and a reference under same conditions. A double cuvette (K) is built up by two standard photometric plastic cuvettes (1 × 1 × 4.3 cm). Both contain electrode arrays (E_1_, E_2_) consisting each of 4 gold wires (Au 99.9%, Johnson Matthey, Karlsruhe) with a diameter of 0.25 mm, mounted parallel at a distance of 2 mm on a teflon frame. The required sample volume was 1 ml. These electrode carriers are mounted on a stable socket for electric connection and mechanical adjustment (not shown). The cuvettes are enclosed by a hermetically sealable plastic tank (T) with a copper bottom, which is filled at a height of 2 cm around the cuvettes with water for thermal coupling to an outer temperature controlled water bath. The setup is kept under Ar atmosphere throughout the experiment. Thermic control (20 ± 0.1 °C) of the cuvettes is provided by a water thermostat (Haake "G", Karlsruhe-Berlin, Germany) with a sequential home built temperature fine controller, ensuring highly stable working conditions for the electrodes. Once assembled, these components form a mechanically stable unit, with in- and outlets for gas and samples by small teflon hoses (not shown). The assembly is placed in the center of a solenoid (S), consisting of two cylindrical coils with a inner diameter of 16 cm and a height of 7 cm for applying the vertically orientated EMF (B). The coil for the static field component consisted of 300 turns of coated copper wire (diameter 0.5 mm), the other coil was winded above and had 50 turns.

For electric and magnetic shielding the complete setup resides in a grounded double-walled Permalloy box with a total wall thickness of 1 mm. A overall inhomogeneity ≤ 0.3 % of the generated fields was determined inside the box with a triaxial CXM539 magnetometer (CMT GmbH, Herrsching, Germany) over the cuvette locations. For coil calibration the relation of field strength to coil current could be ascertained directly in measurement series with the magnetometer for 0.1–100 μT, showing a overall deviation from linearity ≤ 0.2 % (DC and AC), so currents corresponding to even lower field strengths were obtained by extrapolation.

**Figure 2:**
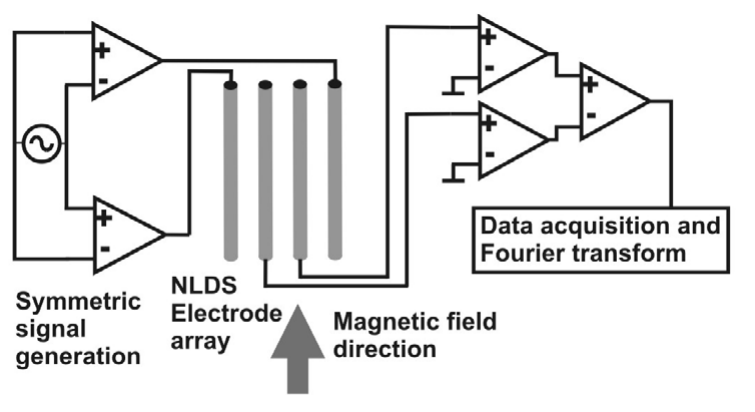
**NLDS measurement setup (schematic). **The voltage control signal is applied by a symmetric amplifier to the outer two of a plane 4 gold electrode array. The NLDS signal generated by the sample is clamped by the inner two electrodes. It will also be preamplified symmetrically, digitized by a fast computer controlled analog-digital converter and fourier analyzed by the data acquisition software. The static and dynamic magnetic field component is directed parallel to the electrode plane. The measuring station provides two such NLDS setups, enabling a simultaneous examination of two samples under same conditions.

Signal processing was mostly done as previously described [27]. Figure 2 shows the schematic circuit diagram of the special NLDS measurement setup used here: The sinusoidal controlling voltage (100–1100 mV) for NLDS with a frequency of 2 Hz was applied to the two outer electrodes by a symmetric amplifier (output impedance 50 Ω). The inner two electrodes were connected to the input ports of a differential preamplifier. Because a simultaneous examination of two samples under same conditions is required, a second identical electrode array with preamplification must be available. The resulting signals were digitized by a computer controlled multi channel DA/AD-converter (Lab-PC+, National Instruments, Austin TX U.S.A.). This board also supplied the voltages for the NLDS and the control of the EMF. A function generator (Krohn-Hite Model 5200) generated the sine curve for the AC magnetic field with a frequency accuracy of 0.1 %. The two operational power amplifiers of a OPA 2541 chip drove the solenoids generating the constant as well as the variable magnetic field components, which were monitored by the coil currents and the magnetometer.

For cleaning, the electrodes were first treated with chromosulfuric acid for 1 h at room temperature and intensively rinsed with de-ionized water. This procedure was repeated approximately once per week. An improved long-term electric stability was obtained by slight modifications of the treatments described by Woodward *et al. *[23] and Yardley *et al. *[28]: The electrodes were additionally washed with chloroform, sonicated for 20 min in a detergent solution (0.5 % Triton X-100 in water), treated with CaCl_2 _(0.5 M in water) in a ultrasonic bath (Bachhofer, Reutlingen), and finally rinsed with de-ionized water (<2 μS). This treatment resulted in amplitude deviations ≤ 5% over an experimental session of up to 2 h. If electrodes were not used for DC measurements, but for NLDS, they were additionally coated with a thin polymer film in order to improve noise reduction and stability [24].

### Measurement techniques

The cuvettes could be charged with the test solutions, discharged and rinsed through the teflon hoses by a syringe. A sample volume of 1 ml was used. Device specific, systematic errors were routinely checked by exchanging the electrode arrays used for sample and reference measurements and testing several cuvettes of the same type. After loading they were flooded with Argon for about 10 min. in order to remove O_2 _from the solutions, avoid oxidation reactions and subsequent arising of reactive oxygen species (ROS) in the solute, then the hoses were sealed with rubber caps. After reaching a stable temperature of 20 ± 0.2°C, measurements were started. First 10 "dummy" scans were performed, in order to obtain a dynamic equilibration of the electrodes. *B_dc _*= 40 μT was selected as static magnetic field component for the ICR condition, because it is of comparable intensity as the natural geomagnetic field of the earth. A new sample was used for every experiment, an "aging effect" of the test solutions was observed, similar to an earlier seen effect, which resulted in a decreasing reproducibility for experiments with magnetic field exposed lipid vesicles [27].

Three types of techniques for measuring the electric currents in the solutions were applied, always using the gold electrode array described above:

1) For the validation of the ICR parameters of the Glu-HCl solution, the experiment of Zhadin *et al. *[25] was repeated. The DC voltage of 80 mV was applied to the outer electrodes (+40 mV and -40 mV), and the current through the solution was calculated from the resulting voltage between the inner electrodes. The current calibration was earlier performed with 10 mM HCl and the Glu-HCl solution. By that way, used by many established voltammetric techniques [29], superimposing electrode transition potentials can be widely avoided, in contrary to a direct current measurement with a two electrode system. A constant magnetic field *B_dc _*= 40 μT or 50 μT and a frequency sweep of the alternating magnetic field *B_ac _*= 50 nT (parallel to *B_dc_*) from 2 to 7 Hz with 0.025 Hz/s and a resolution of 0.05 Hz were used.

2) For the investigation of the ICR transition with NLDS the same magnetic field setup is used like described under 1), the NLDS sine wave was applied on the electrodes (instead of the DC-voltage) and a constant magnetic field *B_dc _*= 40 μT was used.

3) Finally the FRV setup allowed the frequency analysis of the electric signals with variable amplitudes using the DNLDS technique described above. Glu-HCl samples were exposed to constant ICR conditions (*B_dc _*= 40 μT and *B_ac _*= 50 nT, 4.14 Hz fixed), for reference experiments only the static component (*B_dc _*= 40 μT) was applied with *B_dc _*switched off. The amplitude of the sinusoidal scanning voltage was increased in each experiment from 100–1100 mV in steps of 10 mV, record by record, the duration of each cycle was 4 s. The two data sets (from Glu-HCl and HCl sample) yielded by every single record were seperately Fourier transformed in order to get the spectra, these two spectra were divided by themselves (Glu-HCL spectrum by HCl spectrum) and the ratio spectrum was subsequently attached to a data file on a harddisc for later evaluation.

## Results

### Exploring Zhadin et al.'s experiment

**Figure 3:**
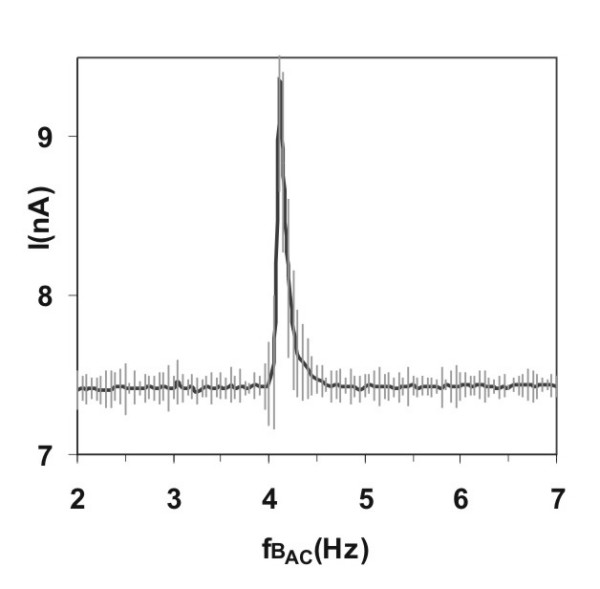
**Current increase at ICR (DC). **Current increase through the glutamic acid /HCl solution (2.24 mM, pH = 2.85) at and near ICR conditions. The static magnetic field strength is *B_dc _*= 40 μT, the amplitude of the alternating field *B_ac _*is 50 nT, the frequency resolution Δf = 0.05 Hz. Course using a constant electrode voltage of 80 mV ("Zhadin's experiment").

Applying a constant magnetic field of *B_dc _*= 40 μT at pH 2.85 and scanning the alternating magnetic field *B_ac _*from 2–7 Hz in steps of 0.05 Hz, a sharp peak was observed at 4.15 Hz. The peak current is about 20% larger than the mean ionic current of 7.4 nA, the peak width at half-height is 0.3 Hz (Figure 3). Equation (1) was validated by repeating the experiment ten times at an altered static magnetic field strenght of *B_dc _*= 50 μT. The current peak shifted to 5.2 ± 0.05 Hz with a height of 9.08 ± 0.3 nA, which lies approximately 22% over the mean ionic background current. These data verify the results of Zhadin *et al. *[25], and the field-dependence is in agreement with Eqn. 1. The signal was observed over a concentration of 2–10 mM. The signal became too small at c_Glu _< 2 mM, and there was insufficient solubility c_Glu _>10 mM (at 20°C). Subsequently, the pH-dependence was determined under identical magnetic field and scanning conditions mentioned above. Resonance effects are only seen in a narrow pH range of Glu-HCl (pH 2.75 – 2.90), with an maximum at 2.85, and vanishes outside this range.

After this verification of the experiment of Zhadin *et al. *[25], these electric measurements were accompanied by some UV-VIS light scattering investigations, which should give information about possible colloidal properties of the sample. Glu-HCl solutions were investigated at a wavelength of λ = 260 nm with the pH adjusted from pH 2.55 to 3.25, showing a significant scattering maximum around pH 2.8 (data not shown).

Further some DC voltage scans were performed with the gold electrode array for Glu at pH 2.85, and for dilute HCl adjusted to pH 2.85, applying only a static magnetic field *B_dc _*= 40 μT (no *B_ac_*). A voltage range of 100–1000 mV was selected to allow a comparison with the voltammetric information out of the frequency resolved voltammetry (FRV). Again, maxima of conductivity were obtained, they lie at 250 ± 10 mV for Glu-HCl and 280 ± 10 mV for water/HCl pH 2.85 (data not shown).

### NLDS spectroscopy

**Figure 4:**
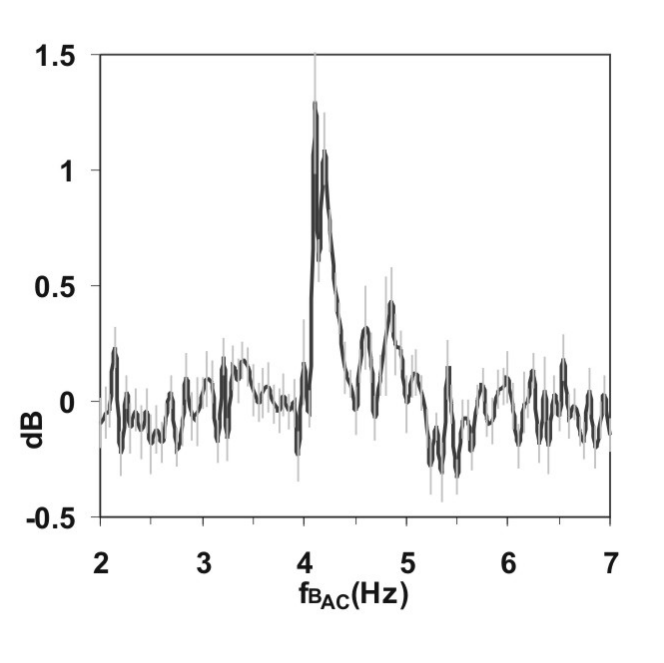
**Current increase at ICR (AC). **Course of the 2^nd ^harmonics of NLDS spectra taken for every scanned frequency of *B_ac_*. Data were related to reference scans with *B_dc _*= 40 μT, but without *B_ac_*. The grey bars indicate standard deviations. Other conditions like Figure 2.

**Figure 5:**
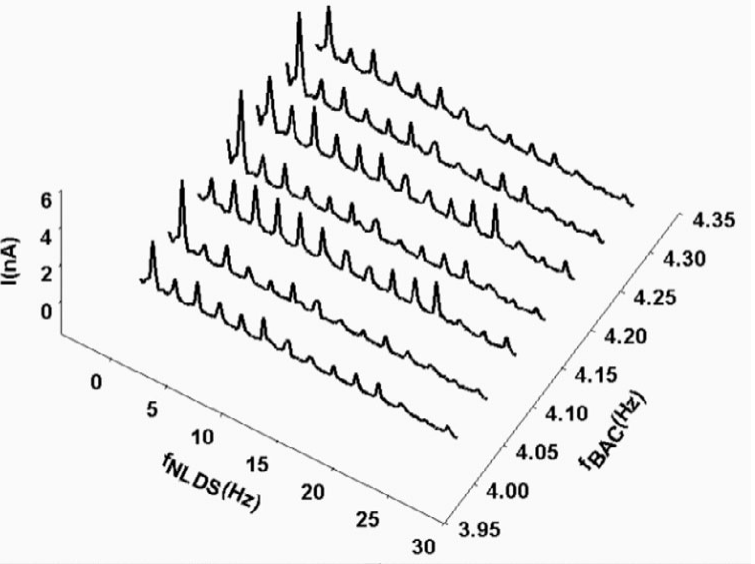
**NLDS spectra on ion cyclotron resonance (ICR) transition. **3D-representation of the NLDS resolved current through a glutamic acid / HCl solution (2.24 mM, pH 2.85) during transition of the ICR condition (static magnetic field *B_dc _*= 40 μT, alternating field *B_ac _*= 50 nT, f_BAC _= 4.14 Hz) in steps of 0.05 Hz.

Next, the solutions were investigated by NLDS spectroscopy, in order to investigate in which way the frequency composition of the current spectra will change, when the predicted ICR condition for Glu-HCl is matched (*B_dc _*= 40 μT and a *B_ac _*with *f *= 4.15 Hz). 15 experiments were performed and averaged. Figure 4 shows the power of the 2^nd ^harmonic (referenced against dilute HCl, pH 2.85). The full dataset is shown in Figure 5 on an absolute current scale, for magnetic frequencies of 4.00–4.30 Hz in a 3d-representation. The 1^st ^harmonic is split up into 2 closely spaced peaks around the ICR frequency. This is also well seen in Figure 4, an effect not seen in the "Zhadin's" DC experiments [25] without frequency resolution. Furthermore an increase of the 2–6 harmonics is seen in Figure 5 for 4.10 and 4.20 Hz magnetic frequency, closely flanking the ICR value. The average standard deviation of these experiments was 8.2 % of the average Power of all DNLDS spectra.

### Kinetics

The following kinetic experiment should clarify, in which way the conductivity of the Glu solution is affected by repeated transitions through the ICR conditions. 12 experiments were performed, each with a new Glu-HCl sample.

**Figure 6:**
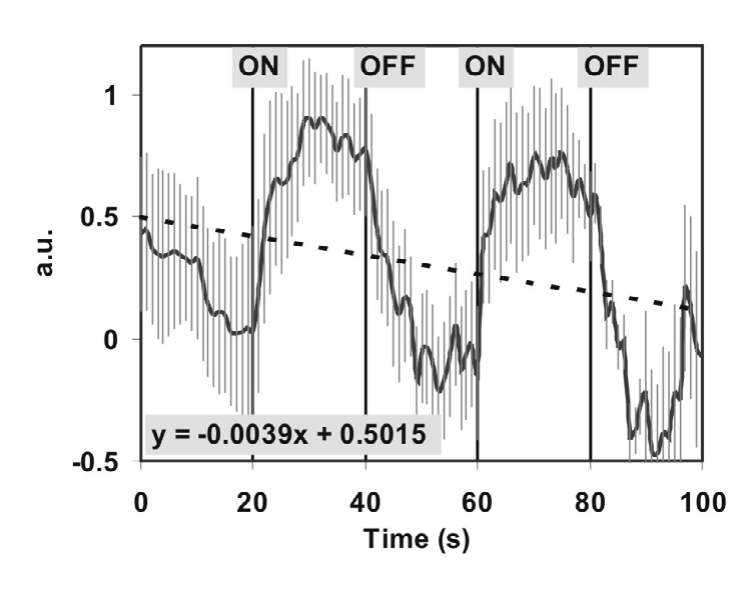
**Current kinetics of switched ion cyclotron resonance (ICR) condition. **Kinetics in arbitrary units (a.u.) of the ICR condition to a glutamic acid / HCl solution (2.24 mM, pH 2.85). The static magnetic field *B_dc _*(40 μT) was applied permanently, the alternating field *B_ac _*(50 nT, 4.14 Hz) was applied as indicated by "on" and "off". One experiment consists of a set of 101 DNLDS spectra performed by a 2 Hz sinus signal with 100 mV Amplitude. The data first 5 harmonics (2, 4, 6, 8, 10 Hz) where normalized and then averaged. Data are calculated out of 12 independent experiments. The grey lines mark the standard deviations, the dotted straight line shows the linear regression of the negative drift, represented by the equation **y = -0.0039t + 0.5015**.

100 DNLDS spectra were recorded with single 2 Hz sinus signals with 100 mV amplitude. *B_dc _*= 40 μT was permanently applied in all experiments, while *B_ac _*with *f *= 4.15 Hz was applied only during measurement no. 20–39 and 60–79. Subsequently, the courses of the lowest 5 harmonics (for 2, 4, 6, 8 and 10 Hz) were normalized to ± 1, and all 12 experiments were averaged, Figure 6 therefore represents the kinetics averaged over a total of 60 datasets. Because of the standardization, data are scaled in arbitrary units (a.u.). The power difference between "on" (exposure) and "off" periods is 1.38 ± 0.34 dB, standard deviations are drawn as bars.

Changes of the signal intensity become obvious, when switching the alternating magnetic field on or off. Over the entire experiment there seems to be a constant drift which we take as an indication for irreversible processes. This drift is indicated by the dotted line, which results from a linear regression of the entire dataset (-0.0039*t *+ 0.5015). The course seems to reach a new steady value after on/off switching of the alternating magnetic field with a time delay, which seems larger, when ICR is switched off. The average current change after the switching processes is -0.2 nA/s, the negative values result from comparison with a reference.

### Differential NLDS experiments with variable control voltages (FRV)

Finally, the FVR method should show the intensity distributions of the harmonics of the DNLDS spectra and their dependence from the used amplitude of the electrode input voltage. Again Glu-at pH = 2.85 was investigated, using diluted HCl (pH 2.85) as the reference. The ratio of the resulting two NLDS power spectra was calculated according to equation (2), resulting in a logarithmic DNLDS spectrum. 101 such scans (4 s each) were performed for every single experiment, during which the amplitude of the applied course of 4 periods of a 2 Hz sine voltage increased from 100 to 1100 mV in 10 mV steps. Corresponding datapoints of the successive single DNLDS spectra generated one AC voltammogram each, for the respective frequency. Altogether, a set of 201 frequency resolved voltamogramms was obtained, because every spectrum contains 201 data values. Subsequently 20 such experiments were performed in which the solution was exposed to ICR conditions, alternating with 20 experiments, were only the static field was applied (*B_dc _*= 40 μT), but not *B_ac_*. Each of the two groups of experiments were averaged separately. Then the two resulting datasets were subtracted (ICR experimental data minus data of the experiments with ICR condition switched off). This differential dataset had a total amplitude of 2.03 ± 0.38 dB, presenting just the contribution of Glu, because the voltammetric background from HCl was subtracted.

**Figure 7:**
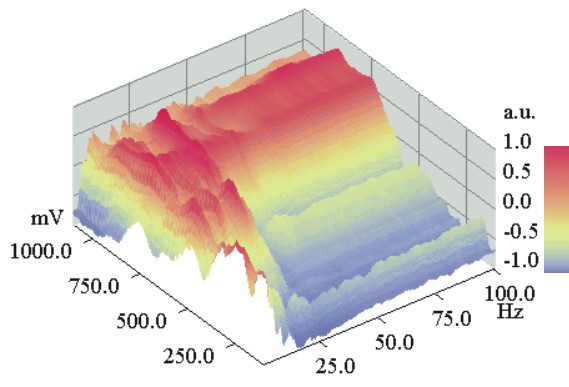
**DNLDS resolved voltammogram dataset (normalized to spectral axis):**Normalizations of the DNLDS resolved voltammogram dataset (sinewave 2 Hz with amplitude rising from 100–1100 mV, details of gaining data see text) of a Glutamic acid / HCl solution (2.24 mM, pH 2.85) under ICR Conditions (B_dc _= 40 μT, B_ac _= 50 nT, 4.14 Hz). Datapoints are colored resp. shaded according to the scale on the right border. Normalization of the spectra for each Amplitude shows a rising proportion of higher frequencies with a local (at about 500 mV) and a total maximum (at about 700–800 mV). By contrast, the proportions of the base frequency (2 Hz) and the lower harmonics decline.

**Figure 8:**
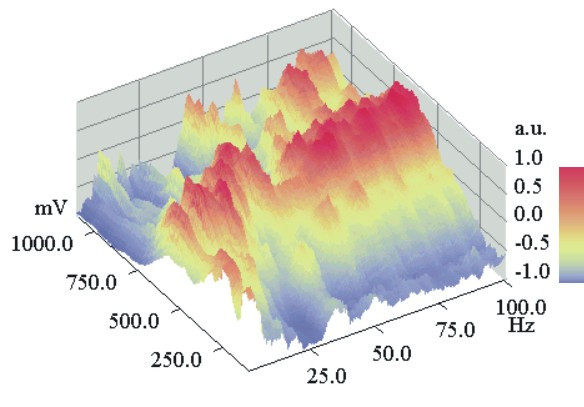
**DNLDS resolved voltammogram dataset (normalized to voltage axis):**The same dataset and representation style like Figure 9, but with normalization of the single voltammograms for each frequency. For low frequencies (<5 Hz) Voltammograms have a maximum at about 250 mV, comparable to the pure DC volt scans. But with rising spectral harmonics voltammetric maxima occur at about 700 mV with overlaying intensity patterns of 4 and 16 Hz in distance. Worthy of remark are 62, 78 and 94 Hz, these all are four folds of the used base ICR resonance frequency 4.14 Hz.

**Figure 9:**
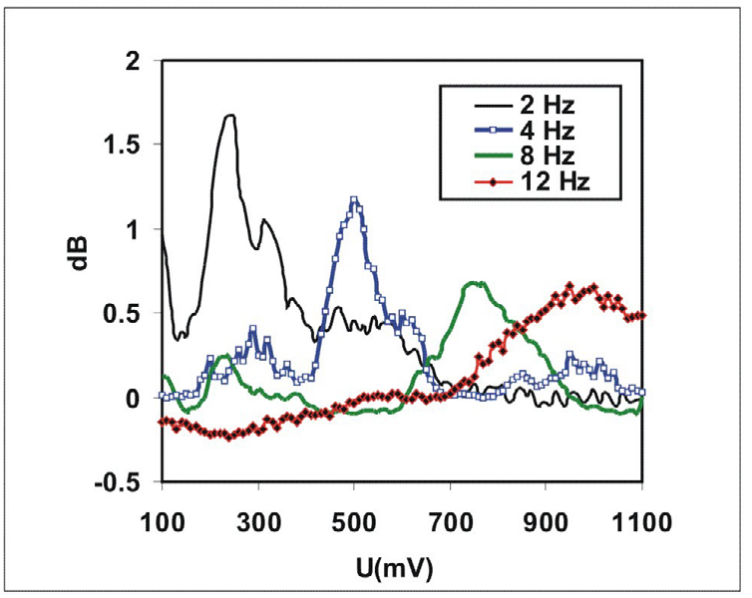
**Extracted voltage courses of the DNLDS resolved voltammogram dataset. **Voltammograms for some harmonics of the DNLDS resolved voltammogram dataset (sine wave 2 Hz with variable amplitude 100–1100 mV, not normalized here, see text for details) of a glutamic acid / HCl solution (2.24 mM, pH 2.85) under ICR Conditions (B_dc _= 40 μT, B_ac _= 50 nT, 4.14 Hz).

Subsequent data normalization should allow a better comparison of spectra recorded with different amplitudes and likewise of voltammograms at different frequencies. Therefore in Figures 7, 8 the full dataset is shown, again after standardization in a range from – 1 to 1. Figure 7 presents the data with standardization on the voltage axis for the voltammograms belonging to the individual frequencies. Figure 8 contains the same dataset, but with standardized spectra. The intensity maximum shifts with rising frequency from approx. 250 mV to 500 mV for frequencies <40 Hz, it then remains constant around 500–700 mV for higher frequencies. So most information will be contained in the low harmonic orders. Figure 9 shows the voltage dependent behaviour at the NLDS fundamental frequency (2 Hz) and three harmonics in the lower range (4, 8, and 12 Hz). Broad maxima are obvious, which seem to shift to higher voltages with increasing harmonic order by about 60 mV/Hz. The intensities increase to a local maximum at approx.25 Hz. At higher frequencies, the amplitude effects caused by the exposure to ICR conditions have a local maximum at 480 mV and merge into a continuum beyond 750 mV for all higher frequencies, according to the predominating capacitive damping of aqueous solutions with rising frequency.

## Discussion

All results suggest the existence of a sensitive magnetic field effect on the conductance of a aqueous Glu solution. The effect shows no linear dependency of magnetic field parameters, it is rather peaking in a narrow range of combinations of static magnetic field strengths and frequencies of additional alternating magnetic fields, described by Eqn. 1.

Several precautions were applied, in order to avoid artefacts as best as possible. So it has been shown, that the signal to noise ratio will be improved significantly by clamping the voltage drop inside the electrolyte and, if needed, by a subsequent calculation of the current by calibration functions, instead of a direct current measurement. These techniques are wide spread in voltammetry [26] and obligatory in NLDS [23]. Because the voltage clamping ideally should work without any electric current flow, the electrode surface transition potentials could more likely be excluded for causing the observed EMF effect ("electrode effects"). It should be least then apply, if a cell voltage is used bellow the electrochemical potentials of the electrode-electrolyte system, and independent from the other experimental setup.

Different explanations are recently discussed for the kind of EMF effect observed here, all of them suppose a non linear oscillator principle described by quantum mechanical terms, allowing energetic interactions with the environment far below the thermal equilibrium of life processes. This search for "wave functions fitting in a properly sized box" should consequently provide an explanation for the repeatedly observed effects of effectiveness windows, regarding specific field strengths and frequencies of the EMF, e.g. seen on green algae grown in a magnetic gradient [30]. Ion channels of biological membranes were proposed in a early work of Liboff [31] for a suitable environment supporting ICR. A model of Binhi *et al. *[14,32] is based on an interference mechanism of quantum states of ions within protein cavities. The quantum dynamic description of an ion is given for the case of ion-protein complexes that rotate in magnetic fields. The individual molecular rotation is taken into account. The spatial distances considered here are in the size of the molecules involved, cavities built by proteins, and their bond lengths.

A quantum electrodynamic description needing no additional supporting structures like protein molecules or lipid membranes was worked out by Giudice *et al. *[33], as an attempt to explain the experimental results of Zhadin *et al. *[25]. It is based on an underlying two-phase domain model of the solvent water, in which at room temperature ~40% of its volume is organized in spheres with a diameter of approximately 100 nm providing coherence for the included water molecules. These spheres should establish a stable frontier region with a thickness of ~4 nm, which allows a undisturbed ion movement, separated by an energy gap 0.26 eV against the surrounding, non coherent water phase. The circulation frequency of the ions in the frontier region should be given by equation (1) and be dependent on the external magnetic field strength. An additional superimposed alternating field *B_ac _*with the same frequency will modulate the radii of the orbits. As a consequence, the ion orbits fit no longer the frontier region and the ions escape into the surrounding water phase, where they increase the conductance. This model also tries to describe the results of [25] quantitatively, but takes therefore in account the electrode geometry of the original experiment.

Further attention should turned to the comparably long persistence time of the ICR state (see Figure 6) implying a comparable long lifetime. Considering the existence of supramolecular orders of liquid water, such long lifetimes (>10 s up to hours) have been predicted for these states sensitive to weak EMF at biologically relevant temperatures. Ponomarev *et al. *[34] propose linearly ordered chains and clusters like a liquid crystal phase in water which interact with EMF. The soliton theory was applied for description. Studies on the electromagnetic "memory effect" of water implicate even high sensitivity and long lifetimes [35], and are probably caused by the same mechanism as the effects observed here. An more hypothetical two phase model also providing boundary layers has been emphasized by Colic *et al. *[36]. The authors discuss the presence of micro-dispersed gas bubbles. But this possibly can be discarded more than likely in our experiments, because degassed solutions were used throughout.

Special attention deserve the obvious frequency dependent amplitude windows of the dielectric currents, which are observed in the NLDS experiments (FRV) with variable amplitudes. Two explanations for this effect would be possible. The additional electric field caused by the AC signal of the NLDS could modulate the charged particles inside a "quantum box", whatever will be the reason for its existence. An indication for such a mechanism could be the more or less ordered local maxima of conductivity in spectral as well as in the voltammetric domain of the data. But the frequency dependent conductivity band shifts of the FRV experiments (Figure 9) could either result from a "simple" interference with the frequency of the *B_ac _*field, which can be tuned on its part in discrete multiples, corresponding to possible "overtones" of the ICR (orbital) frequency of the ions. Interactions of the internal electric and external magnetic field could probably cause side band modulations. They are probably responsible for the seen splitting up of the ICR resonance peak (figure 4) when using a AC instead of a DC probe voltage.

For progressed investigation of the observed effects some additional properties of the electric charge environment of the Glu ion should be known. The isoelectric point of Glu is at pH 3.22, the pK of the α-COOH-group is 2.19, that of the β-COOH at 4.25, the small optimum for the EMF effect around pH 2.85 does not coincide with any of these points. The Debye-Hueckel radii for Glu are about 5 nm, they determine the free ion movement, and influence consequently the current. Moreover, they could be responsible for a proper fit of the spatial ion distribution to the environing structure whatever, which enables a resonant EMF effect.

## Conclusion

The results strengthen the idea, that weak electromagnetic fields can cause an resonance effect on molecular or even supramolecular scale in electrolyte solutions [33,35], and thereby possibly, influence biological processes, which involve these electrolytes. In this work, the electric currents in a glutamic acid solution were investigated with frequency resolution after applying weak EMF. The resonance peaks and the overtone-analysis in response to weak static plus alternating EMF support the existence of the ICR phenomenon in aqueous solutions containing electrolytes. A analysis of the data is possible under the basic assumption of a far reaching principle of arrangement (realized e.g. by the solvent matrix), which allows quantum electrodynamic processes on the nano-physical scale or larger.

In general any kind of a suitable coherence mechanism should be essential for the observed effects in a dense medium like water, which had to support an energy gap against the thermal fluctuations of the environment, and enable a movement of charged particles which are only magnetically coupled to their outer environment. Not at least, the high sensitivity of the ICR to weak electromagnetic fields should be regarded. It makes the modulation of biological processes by the weak EMF of our everyday environment conceivable [37], possibly inducing likewise health risks and chances for new therapies, hardly minded till this day. Especially concerning the earlier [25] and the present study, glutamate is a neurotransmitter and is involved in a couple of other biological processes.

The geomagnetic field, with all its anomalies and regional differences [38], in combination with all the natural and civilizing EMF, overlap with a wide range of possible ICR of biologically relevant ions. But also technical applications basing on the ICR are imaginable, as a potential direction of future research. Its further investigation will be worthwhile, by new experiments, comparing field studies of health phenomena, and not at least a further clear up of its physical principle.

## List of abbreviations

**EMF: **(low frequency) electromagnetic field

**ICR: **ion cyclotron resonance

**NLDS: **non linear dielectric spectroscopy

**DNLDS: **differential non linear dielectric spectroscopy.

**FRV: **frequency resolved voltammetry

**Glu-HCl: **A glutamate solution adjusted to pH 2.85 with hydrochloric acid (HCl).

## Authors' contributions

The author itself carried out all experiments and drafted the manuscript.
